# Is Carboxyhaemoglobin an Effective Bedside Prognostic Tool for Sepsis and Septic Shock Patients?

**DOI:** 10.2478/jccm-2023-0031

**Published:** 2023-11-14

**Authors:** Bianca-Liana Grigorescu, Oana Coman, Anca Meda Văsieșiu, Anca Bacârea, Marius Petrișor, Irina Săplăcan, Raluca Ștefania Fodor

**Affiliations:** Department of Anesthesia and Intensive Care, George Emil Palade University of Medicine, Pharmacy, Science, and Technology of Targu Mures, Romania; Department of Simulation Applied in Medicine, George Emil Palade University of Medicine, Pharmacy, Science, and Technology of Targu Mures, Romania; Department of Infectious Diseases, George Emil Palade University of Medicine, Pharmacy, Science, and Technology of Targu Mures, Romania; Department of Pathophysiology, George Emil Palade University of Medicine, Pharmacy, Science, and Technology of Targu Mures, Romania; County Emergency Clinical Hospital, Targu Mures, Romania

**Keywords:** sepsis, septic shock, SOFA, APACHE II, SAPS II, carboxyhaemoglobin, neutrophil-to-lymphocyte ratio

## Abstract

**Introduction:**

Proper management of sepsis poses a challenge even today, with early diagnosis and targeted treatment being the most important steps. Easy, cost-effective bedside tools are needed in order to pinpoint towards the outcome of sepsis or septic shock.

**Aim of study:**

This study aims to find a correlation between Sequential Organ Failure Assessment (SOFA), Acute Physiology and Chronic Health Evaluation II (APACHE II) and Simplified Acute Physiology Score II (SAPS II) severity scores, the Neutrophil-Lymphocytes Ratio (NLR) and carboxyhaemoglobin (COHb) levels in septic or septic shock patients with the scope of establishing a bed side cost-effective prognostic tool.

**Materials and methods:**

A pilot, prospective, observational, and ongoing study was conducted on 61 patients admitted with sepsis or septic shock according to the SEPSIS 3 Consensus definition. We followed clinical and paraclinical parameters on day 1 (D1) and day 5 (D5) after meeting the inclusion criteria.

**Results:**

On D1 we found a statistically significant positive correlation between each severity score (p <0.0001), r = 0.7287 for SOFA vs. APACHE II with CI: 0.5841–0.8285, r = 0.6862 for SOFA vs. SAPS II with CI: 0.5251–0.7998 and r = 0.8534 for APACHE II vs. SAPS II with CI: 0.7663 to 0.9097. On D5 we observed similar results: a significant positive correlation between each severity score (p <0.0001), with r = 0.7877 for SOFA vs. APACHE II with CI: 0.6283 to 0.8836, r = 0.8210 for SOFA vs. SAPS II with CI: 0.6822 to 0.9027 and r = 0.8880 for APACHE II vs. SAPS II., CI: 0.7952 to 0.9401. Nil correlation was found between the severity scores, NLR and COHb on D1 and D5.

**Conclusion:**

Cost-effective bedside tools to pinpoint towards the outcome of sepsis are yet to be found, however the positive correlation between the severity scores point out to a combination of such tools for prognosis prediction of septic or septic shock patients.

## Introduction

In 2016 the SEPSIS 3 Consensus updated the definition of sepsis and septic shock. Thus, sepsis represents a life-threatening organ dysfunction caused by a dysregulated host response to infection. Moreover, septic shock, a subset of sepsis, is defined by profound circulatory, cellular, and metabolic abnormalities, and a greater risk of mortality than sepsis [1, 2]. Early diagnosis, infection control and aggressive resuscitation are part of the globally standardized management [3,4,5,6].

Early assessment of the severity of sepsis and its prognosis remains imprecise. The scarcity of comparative studies of different prognostic markers such as C-reactive protein (CRP), procalcitonin (PCT) and prognostic scores such as SOFA, Acute Physiology and Chronic Health Evaluation II (APACHE II) and Simplified Acute Physiology Score II (SAPS II) requires further investigation.

Starting with the year 2000, Roman Záhorec studied more closely the ratio between neutrophils and lymphocytes (Neutrophil to lymphocyte ratio - NLR) and its predictive role regarding the evolution of sepsis [7, 8]. It can be easily calculated by dividing the number of neutrophils to that of lymphocytes. Neutrophils contribute to the innate immune response through phagocytosis and the release of cytokines whilst lymphocytes, representing the adaptive immune response, decrease in number under stress conditions. Inflammation is due to demarginating and accelerated apoptosis [8].

Neutrophilia and lymphocytopenia are reactions to physiological stress, thus NLR represents the balance between the innate and adaptive immune response [8, 9]. However, NLR cannot pinpoint the exact cause of the patient’s condition, high values are found in severe inflammation, trauma, major surgery, neoplasm, being associated with high mortality and morbidity [3, 8]. NLR is increased by any source of physiologic stress and might be used in sorting the “sick versus not sick” [9].

Optimal cut-off values for measuring stress intensity and inflammatory response have been refined according to clinical studies and observations. The normal cut-off value of NLR is approximately 1–3, the values increase in proportion to the degree of physiological stress, especially in septic shock [9, 10]. Values above 3 and below 0.7 are pathological and are associated with significant mortality and morbidity [8]. The gray area, which corresponds to an NLR of 2.3 – 3.0, raises the suspicion of a latent, subclinical inflammation. Values between 3 and 17 represent different degrees of inflammation; septic shock is found between 17 and 23, while critical systemic inflammation, terminal cancer, major surgical interventions, polytrauma correspond to NLR ≥ 23+ [8, 9].

The role played by the liver in sepsis has been thoroughly studied across the years, the inhibition of hepatocyte clearance of bilirubin and elevation of transaminase levels mirror the intensity of liver function impairment [11]. The matter in question is represented by the large amount of carbon monoxide (CO) produced by the liver in sepsis, by catabolism of heme via hemeoxygenase-1 (HO-1) pathway. CO is detected in blood as carboxyhemoglobin (COHb) and CO excretion in breath [12].

Therefore, COHb levels might be utilized as a bedside prognostic tool to monitor the progression of sepsis and could provide early information regarding the outcome of both bacterial and viral infection [13].

APACHE II, SOFA and SAPS II scores are well-known mortality predictors calculated for septic patients in the Intensive Care Unit (ICU) to assess disease severity, treatment response, and mortality risk [10, 14].

This study aims to find an easy, quick, bed side and cost-effective tool for predicting sepsis and septic shock outcome by correlating the SOFA, APACHE II and SAPS II severity scores with NLR and COHb levels, in order to guide the clinician on the evolution of the disease.

## Materials and methods

**Fig. 1.**
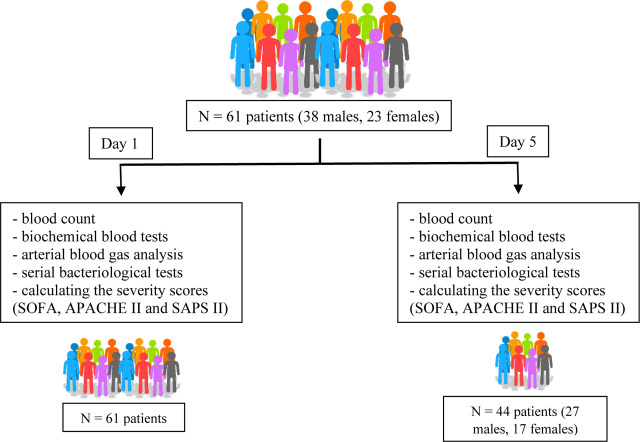
**Visual description of the study** (N – number of patients; SOFA - Sequential Organ Failure Assessment; APACHE II - Acute Physiology and Chronic Health Evaluation II; SAPS II - Simplified Acute Physiology Score II)

This is a pilot, prospective, observational, and ongoing study conducted on a number of 61 patients admitted to the Anesthesia and Intensive Care Department of the Târgu Mureș Emergency Clinical County Hospital, Mureș County, Romania between July 2021 and September 2022 (Figure 1).

The inclusion criteria were: age above 18 years and diagnosis of sepsis or septic shock according to the SEPSIS 3 Consensus. The exclusion criteria were: current neoplasia, current chemo- or radiotherapy, corticosteroid treatment or immunosuppressive medication, or evidence of autoimmune disorders.

Patient data were obtained on Day 1 (D1) and Day 5 (D5) after meeting the criteria of diagnosis of sepsis or septic shock. Clinical and paraclinical parameters followed were: blood count, biochemical blood tests, arterial blood gas (ABG) analysis, serial bacteriological tests, along with calculating the severity scores (SOFA, APACHE II and SAPS II). The need for vasoactive medication, along with the ventilation parameters were recorded. COHb was determined by an arterial puncture using a standard heparinized syringe (Stat Profile Prime Plus, Manufacturer: Nova Biomedical, Waltham, MA 02454-9141 USA, year of manufacture 2018). All the obtained data were recorded in a database.

Regarding the interpretation of Neutrophil to lymphocyte ratio presented in our study, we used the NLR-meter implemented by Roman Záhorec [8].

This study was conducted with the approval of the Hospitals Ethics Committee approval no 5416/25.02.2021 for septic patients. The General Data Protection Regulation (GDPR) agreement was respected, and the obtained data was used for research purposes only.

### Statistical Analysis

The obtained data were recorded in a database and statistically analyzed using GraphPad Prism 8. Data series normality was tested using the D’Agostino & Pearson test. Descriptive statistics are reported as median, minimum, maximum, percentiles (25th, 75th) and interquartile range. For each day (D1 and D5), we performed correlation analysis (Pearson and Spearman two-tailed correlation test) between each severity score (SOFA, APACHE II and SAPS II), NLR and COHb values. All statistical tests used a significant threshold of p = 0.05.

## Results

**Figure 2.**
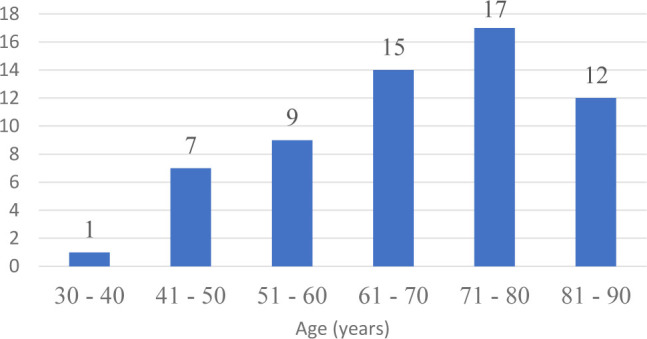
Distribution by age

**Figure 3.**
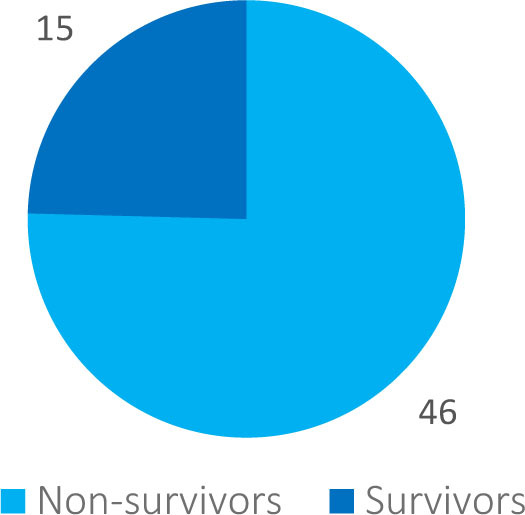
Distribution by mortality

The average age of patients was 68 years, minimum age was 33 years, maximum age 90 years old (Figure 2). The majority of cases were situated in the 60 – 80 years interval, particularly due to decreased immunity and the presence of comorbid disorders, increasing the frailty of patients. The distribution by gender compiled 23 females and 38 males. Out of the 61 patients included in this study, on the first day of enrollment 24 presented with septic shock and 37 with sepsis, and of all the patients 15 survived (Figure 3).

Descriptive statistics for SOFA, APACHE II, SAPS II, NLR and COHb on D1 are displayed in Table 1.

**Table 1.** Descriptive statistics on D1

	**SOFA**	**APACHE II (points)**	**SAPS II (points)**	**NLR**	**COHb**
Minimum	1.000	7.000	17.00	0.7420	0.000
25% Percentile	6.000	14.00	34.50	8.236	0.4250
Median	9.000	22.00	56.00	14.14	1.100
75% Percentile	13.00	30.00	72.00	25.01	1.500
Maximum	19.00	44.00	118.0	59.67	2.500
Range	18.00	37.00	101.0	58.93	2.500
Mean	9.459	22.30	54.61	17.06	1.080
Std.Deviation	4.700	9.426	21.84	11.76	0.6356
Std. Error of Mean	0.6017	1.207	2.796	1.505	0.08206

SOFA – Sequential Organ Failure Assessment; APACHE II – Acute Physiology and Chronic Health Evaluation II; SAPS II – Simplified Acute Physiology Score II; NLR – Neutrophil-Lymphocytes Ratio; COHb – Carboxyhaemoglobin; D1 – day 1

On D1 we found a statistically significant positive correlation between each severity score (p <0.0001), r = 0.7287 for SOFA vs. APACHE II with CI: 0.5841–0.8285, r = 0.6862 for SOFA vs. SAPS II with CI: 0.5251–0.7998 and r = 0.8534 for APACHE II vs. SAPS II with CI: 0.7663 to 0.9097.

**Fig. 4.**
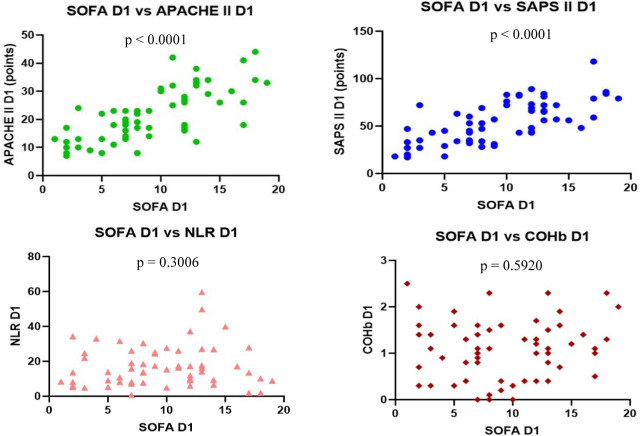
**Positive correlation between SOFA vs. APACHE II and SOFA vs. SAPS II. No correlation between SOFA vs. NLR and SOFA vs. COHb levels on D1** (SOFA – Sequential Organ Failure Assessment; APACHE II – Acute Physiology and Chronic Health Evaluation II; SAPS II – Simplified Acute Physiology Score II; NLR – Neutrophil-Lymphocytes Ratio; COHb – Carboxyhaemoglobin; D1 – day 1)

We found no correlation between the severity scores vs. NLR and COHb levels, however, we found a statistically significant negative correlation between NLR and COHb levels with a CI: −0.4815 to 0.0044956, r = −0.2543 (Figure 4 – Figure 7).

**Fig. 5.**
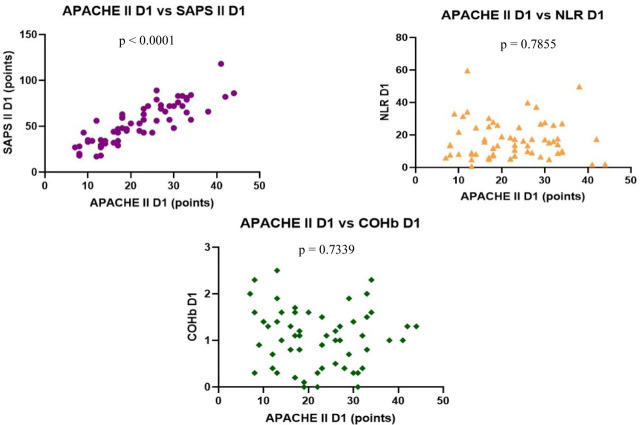
**Positive correlation between APACHE II vs. SAPS II. No correlation between APACHE II vs. NLR, APACHE II vs. COHb levels on D1** (APACHE II – Acute Physiology and Chronic Health Evaluation II; SAPS II – Simplified Acute Physiology Score II; NLR – Neutrophil-Lymphocytes Ratio; COHb – Carboxyhaemoglobin; D1 – day 1)

**Fig. 6.**
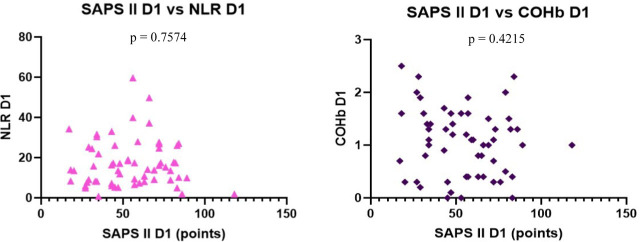
**No correlation between SAPS II vs. NLR, SAPS II vs. COHb levels on D1** (SAPS II – Simplified Acute Physiology Score II; NLR – Neutrophil-Lymphocytes Ratio; COHb – Carboxyhaemoglobin; D1 – day 1)

**Fig. 7.**
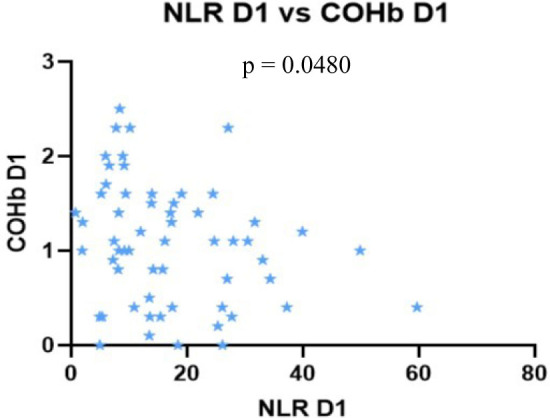
**Negative correlation between NLR vs. COHb on D1** (NLR – Neutrophil-Lymphocytes Ratio; COHb – Carboxyhaemoglobin; D1 – day 1)

Descriptive statistics for SOFA, APACHE II, SAPS II, NLR and COHb on D5 are displayed in Table 2.

**Table 2.** Descriptive statistics on D5

	**SOFA**	**APACHE II (points)**	**SAPS II (points)**	**NLR**	**COHb**
Minimum	0.000	5.000	15.00	0.7860	0.000
25% Percentile	2.000	11.00	29.00	5.422	0.600
Median	7.000	18.00	46.00	9.005	1.000
75% Percentile	12.00	27.00	61.00	17.87	1.500
Maximum	19.00	34.00	85.00	46.75	17.00
Range	19.00	29.00	70.00	45.96	17.00
Mean	7.359	18.74	45.36	13.03	1.471
Std. Deviation	5.334	9.318	20.40	10.84	2.550
Std. Error of Mean	0.8541	1.492	3.267	1.714	0.3982

SOFA – Sequential Organ Failure Assessment; APACHE II – Acute Physiology and Chronic Health Evaluation II; SAPS II – Simplified Acute Physiology Score II; NLR – Neutrophil-Lymphocytes Ratio; COHb – Carboxyhaemoglobin; D5 – day 5

On D5 we observed a statistically significant positive correlation was found between each severity score (p <0.0001), with r = 0.7877 for SOFA vs. APACHE II with CI: 0.6283 to 0.8836, r = 0.8210 for SOFA vs. SAPS II with CI: 0.6822 to 0.9027 and r = 0.8880 for APACHE II vs. SAPS II, CI: 0.7952 to 0.9401.

**Fig. 8.**
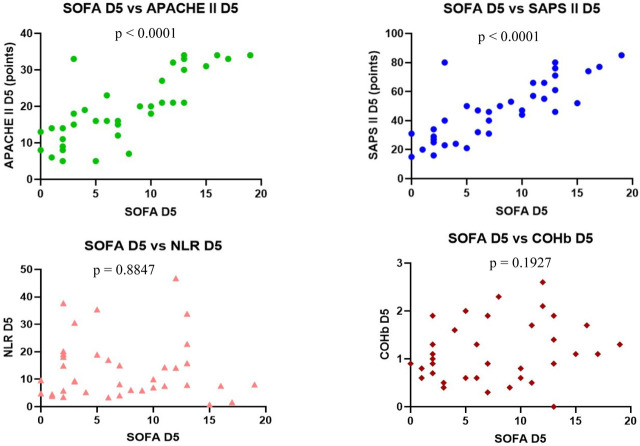
**Positive correlation between SOFA vs. APACHE II, SOFA vs. SAPS II. No correlation between SOFA vs. NLR and SOFA vs. COHb levels on D5** (SOFA – Sequential Organ Failure Assessment; APACHE II – Acute Physiology and Chronic Health Evaluation II; SAPS II – Simplified Acute Physiology Score II; NLR – Neutrophil-Lymphocytes Ratio; COHb – Carboxyhaemoglobin; D5 – day 5)

**Fig. 9.**
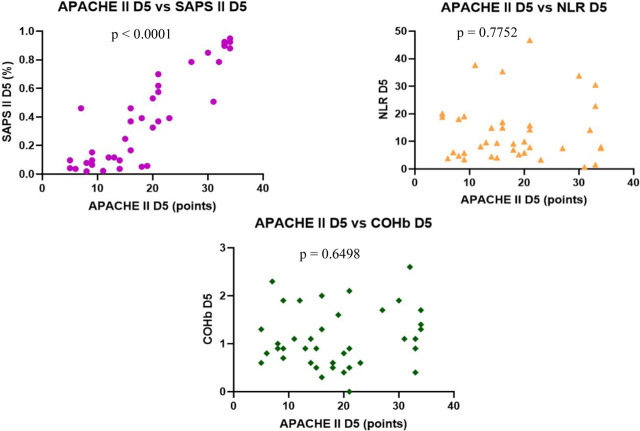
**Positive correlation between APACHE II vs. SAPS II. No correlation between APACHE II vs. NLR, APACHE II vs. COHb levels on D5** (APACHE II – Acute Physiology and Chronic Health Evaluation II; SAPS II – Simplified Acute Physiology Score II; NLR – Neutrophil-Lymphocytes Ratio; COHb – Carboxyhaemoglobin; D5 – day 5)

**Fig. 10.**
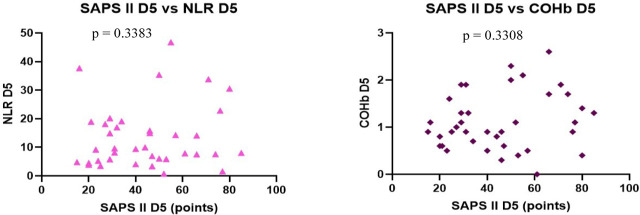
**No correlation between SAPS II vs. NLR, SAPS II vs. COHb levels on D5** (SAPS II – Simplified Acute Physiology Score II; NLR – Neutrophil-Lymphocytes Ratio; COHb – Carboxyhaemoglobin; D5 – day 5)

**Fig. 11.**
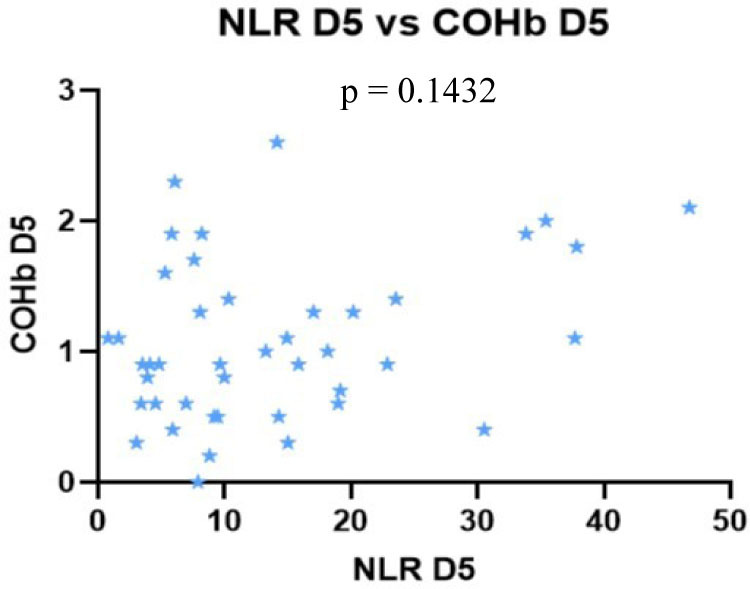
**No correlation between NLR vs. COHb on D5** (NLR – Neutrophil-Lymphocytes Ratio; COHb – Carboxyhaemoglobin; D5 – day 5)

Nil correlation was found between the severity scores, NLR and COHb on D5 (Figure 8 – 11).

In regards of mortality, we evaluated the predictability of evolution of either sepsis or septic shock by observing the changes in NLR and COHb from D1 to D5 and we found the following in Table 3. Survivors of either sepsis or septic shock presented an improvement in the NLR and COHb levels from D1 to D5. In non-survivors we observed a decrease of NLR from D1 to D5 whilst COHb levels increased. Also, in 16 non-surviving patients we managed to record data only on D1 due to death before D5 of study inclusion.

**Table 3.** Predictability of mortality by NLR and COHb variation from D1 to D5 of survivors and non-survivors

	**Survivors (n = 15)**	**Non-survivors (n = 46)**
NLRD1 ↓ D5 and COHb D1 ↓ D5	11	9
NLRD1 ↓ D5 and COHb D1 ↑ D5	1	14
NLRD1 ↑ D5 and COHb D1 ↑ D5	2	3
NLRD1 ↑ D5 and COHb D1 ↓ D5	0	4
NLR only on D1 and COHb only on D1	1	16

NLR – Neutrophil-Lymphocytes Ratio; COHb – Carboxyhaemoglobin; D1 – day 1; D5 – day 5

**Fig. 12.**
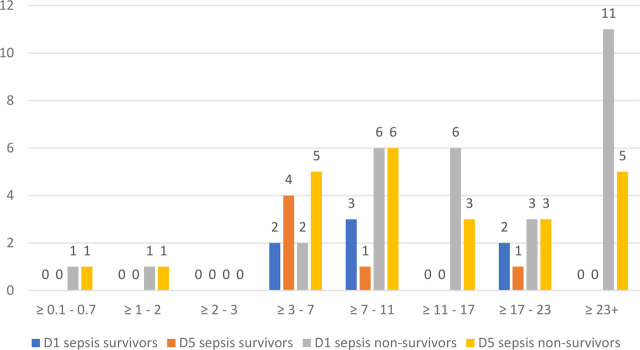
**Evolution of NLR on D1 and D5 in sepsis survivors and non-survivors** (NLR – Neutrophil-Lymphocytes Ratio; D1 – day 1; D5 – day 5)

**Fig. 13.**
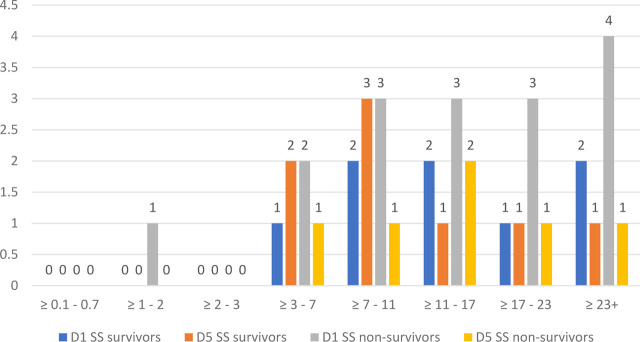
**Evolution of NLR on D1 and D5 in septic shock survivors and non-survivors** (NLR – Neutrophil-Lymphocytes Ratio; SS – septic shock; D1 – day 1; D5 – day 5)

**Fig. 14.**
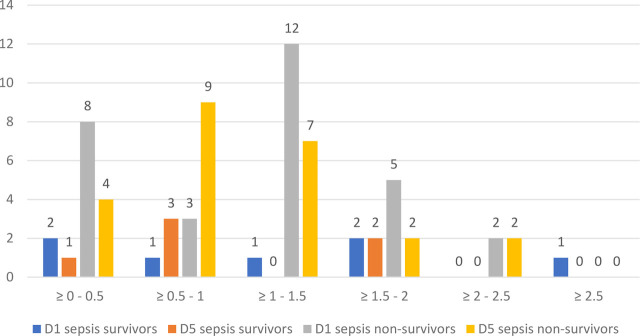
**Evolution of COHb (%) on D1 and D5 in sepsis survivors and non-survivors** (COHb – Carboxyhaemoglobin; D1 – day 1; D5 – day 5)

**Fig. 15.**
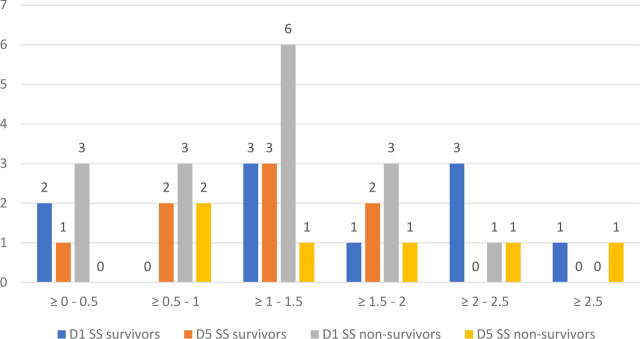
**Evolution of COHb (%) on D1 and D5 in septic shock survivors and non-survivors** (COHb – Carboxyhaemoglobin; SS – septic shock; D1 – day 1; D5 – day 5)

We studied the evolution of both NLR and COHb levels on D1 and D5 for sepsis and septic shock in survivors and non-survivors, and the results are illustrated in Figure 12 – 15.

On D1 of study inclusion, we found that most sepsis non-survivors presented with NLR values over 23, characterized as critical systemic inflammation, maintaining high values in D5 as well. NLR values on D1 of sepsis survivors ranged between 3 and 23, with a decrease towards D5.

On the other hand, septic shock survivors and non-survivors presented high values of NLR throughout D1 to D5, no patients presented values between 0.1 – 0.7 and between 2 – 3, corresponding to the gray area, latent, subclinical, low-grade inflammation.

For COHb, we used increments of 0.5% to categorize the groups of patients, the reference range values from the ABG analyzer were between 0.5 – 1.5%. For sepsis non-survivors we observed that on D1 the majority presented values of COHb between 1 – 1.5%, whilst sepsis survivors were more uniformly distributed between 0.5% and 2%.

Regarding septic shock patients, most non-survivors presented values between 1 – 1.5% in D1 of establishing the criteria for septic shock. In comparison with sepsis survivors, we observed values of COHb in the range of 2 – 2.5% on D1 for septic shock survivors.

A more comprehensive view of the pathology of patients included in the study is achieved by examining the site of infection, cause and the pathogens involved. Regarding the site of infection, the majority of patients included in the study presented pulmonary and abdominal infections (Table 4), hence the predominant aetiology was bronchopneumonia and peritonitis.

**Table 4.** Site of infection and aetiology of infection of patients included in the study.

**Site of infection**	**Number of patients**	**Aetiology**	**Number of patients**
Pulmonary	36	Pneumonia / Bronchopneumonia	33
Abdominal	27	Peritonitis	12
Cutaneous	5	Intestinal obstruction	5
Urinary tract	4	Enterocolitis	2
Bloodstream	3	Sepsis / septic shock of unknown origin	2
Soft tissue	3	Pancreatitis	3
Biliary tract	1	Urinary tract infection	2
Unspecified	1	Cutaneous abscess	5
		Prosthetic joint infection	1

Pathogens involved in the infectious process vary greatly, highest incidence is represented by Acinetobacter baumanii, followed by Klebsiella pneumoniae with its forms and Pseudomonas aeruginosa (Table 5).

**Table 5.** Pathogens associated with infection of patients involved in the study.

**Pathogens**		**Number of patients**
Pseudomonas aeruginosa		9

Klebsiella pneumoniae	CPE	8
	BLSE	2
	unspecified	2

Acinetobacter baumanii		13

Proteus vulgaris		2

Enterococcus faecalis		5

Escherichia coli	BLSE	4
	unspecified	3

Staphylococcus arureus	MRSA	1
	MSSA	2

Streptococcus pneumoniae		1

Candida spp.		8

Clostridiumdifficile		3

Other		16

MDR	Yes	13
	No	48

## Discussions

Proper management of sepsis poses a challenge even today, with early diagnosis and targeted treatment being the most important steps. Easy, cost-effective bedside tools that can pinpoint towards the outcome of sepsis and septic shock have been the pinnacle of research for many years, especially since world economy suffered severe headwinds amid weak growth prospects and heightened uncertainties. [15, 16]. In a fast-paced society, the need for easy-to-read tests should be considered an appendix of physicians.

The severity scores are still a reliable tool used in ICU for assessing the prognostic of either sepsis or septic shock by evaluating disease severity, response to treatment and risk of mortality in the ICU. The importance of appreciating the evolutionary path of pathology plays an important role in effectively prescribing the needed medication. On the first and fifth day of our study we found a positive correlation between each severity score, SOFA, APACHE II and SAPS II, results that are supported by recent scientific literature [14, 17]. However, the debate concerning the accuracy of predicting morbidity and mortality remains, since the scores reflect the state of disease at a certain moment of the patients’ stay in the ICU. Thereby, constant reevaluation of either SOFA, APACHE II or SAPS II, independently or together, ought to be done. Nonetheless, the severity scores do not take into consideration other factors such as lifestyle, received medications or the quality of the follow-up care to estimate in the long run the morbidity and mortality of septic or septic shock patients [18].

Fluid resuscitation and vasoactive agents are part of the treatment of sepsis and septic shock, the impact of hemodynamic instability is also translated as an impairment of microcirculation. Liver dysfunction occurs synergistically with tissue hypoxia and impaired hepatic microcirculation [19]. Production of endogenous CO is the result of heme catabolism by heme oxygenase enzymes [12]. Disruption of heme metabolism and liver dysfunction associated with sepsis leads to an increase of COHb levels mainly due to oxidative stress, hypoxia, cytokines, endotoxins, and inflammatory mediators [13, 20]. COHb is a parameter measured frequently in ICU during routine arterial blood gas analysis, an abrupt change of trend in COHb levels could lead the physician to notice a new course of the disease [12].

Although we observed no statistically significant correlation between COHb level and severity scores, on D1 of meeting the criteria for sepsis, the majority non-surviving patients presented elevated levels of COHb. Septic shock patients, both survivors and non-survivors, on D1 of inclusion in the study, presented elevated COHb levels.

Literature on COHb and the role played in sepsis is scarce, an overview on PubMed returns 34 results from 1974 to 2023. R. Palmieri an V. Gupta published in 2023 a review regarding carboxyhemoglobin toxicity stating that carbon monoxide, after displacing oxygen form hemoglobin, deceases its oxygen-carrying capacity causing tissue hypoxia and acidosis, and plays a role in inhibiting aerobic metabolism by binding to mitochondrial cytochrome oxidase [21].

Another study published in 2023 by G. Vadar and E. Ozek studied COHb levels in late-onset sepsis in pre-term neonates and found increased levels of COHb in the beginning of sepsis that decreased in response to antibiotics, but the variation of COHb, when used in conjunction with other sepsis biomarkers, could predict the outcome of sepsis [22]. Our study found an increase in COHb levels from D1 to D5 in both sepsis and septic shock non-survivors, comparable results to to recent literature [13, 22].

The NLR was introduced as a prognostic tool for sepsis and septic shock because of its simplicity and cost-free [23]. Calculated as a ratio between the neutrophil and lymphocyte counts in peripheral blood, it found its usefulness in predicting outcomes in oncology patients [24].

Drewry et al. found that persistent and profound immunosuppression prior to succumbing to sepsis or septic shock manifests with low count of circulating lymphocytes on the fourth day following the diagnosis of sepsis and could predict short- or long-term survival [25]. Our study identified in D1 in a number of 11 sepsis non-survivor’s values of NLR over 23, corresponding to critical systemic inflammation and supraphysiological stress [7, 8].

Another study, by Buonacera A. et al. suggested that an increase of NLR in under 6 hours following acute physiological stress could suggest the use of NLR as a marker of acute stress before other laboratory parameters, such as C-reactive protein, white blood cell count, etc. [26]. We found increased values of NLR on D1 in 16 patients who succumbed before D5 of the study, suggesting a profound inflammatory status, results found also by a recent metanalysis by Hunag Z. and collaborators, describing that high NLR values are associated with poor prognosis [27]. A loud immune response associated with the presence of cytokine storm, impaired microcirculation causing alteration of mitochondrial function and massive tissue damage are responsible for untimely death [28].

Drăgoescu A. and collaborators, in a study published in 2021, described NLR as a more reliable tool in identifying patients with more severe forms of sepsis when comparing it to the SOFA severity score [4]. In our study we found statistically significant positive correlations in D1 and D5 between each severity score, SOFA, APACHE II and SAPS II, and no correlation between NLR and the severity scores. However, the high values of NLR on D1 for both sepsis and septic shock non-survivors and the correlations we found between the severity scores suggest profound immune system derangement.

CO is an endogenous gas found in exhaled human air, studied since 1920’s when it was attributed to pollution and smoking. Its involvement in inflammation, cell death, and metabolism control is well established [29]. It is mainly produced in the liver by catabolism of heme via HO-1 pathway, along with other products of heme degradation like free ferrous iron and biliverdin [31]. Other sources of CO production are myoglobin, cytochromes, peroxidases, and catalase, contributing 20–25% to the total amount of endogenous CO [30, 32]. CO is detected in blood as COHb and CO excretion in breath. In sepsis, the microcirculation of liver is impaired, hence the intense degradation of heme and increased production of CO. COHb measurement by arterial puncture is a fast, easy and cost-effective method to receive information regarding liver function impairment caused by tissue hypoxia and affected microcirculation. In regard to exogenous CO intoxication, debate is ongoing with studies supporting and combatting the use of COHb as a useful method to detect CO toxicity [29, 30]

Our study had a number of limitations: a small number of patients that were enrolled, as well as being conducted in a single center. This is a pilot and ongoing study involving establishing different bed-side prognostic tools for either sepsis or septic shock.

## Conclusions

NLR and COHb levels are straightforward biomarkers that are easy to calculate and cost-effective that can offer a perspective upon the complex relations of the immune process: inflammation and immunity.

Increased levels of CO produced by the liver, by following the variation of COHb and not the cut-off values, should alert the physician regarding worsening of the condition, even if the patient might be a smoker and have higher COHb values per se.

A combination of prognostic tools should be utilized when aiming to predict the evolution of sepsis or septic shock. Future in-depth studies should focus on identifying the power of NLR and COHb levels as mortality predictors regarding sepsis, as well as considering other easily available biomarkers.
